# Primary Care Physicians’ Satisfaction With Interoperable Health Information Technology

**DOI:** 10.1001/jamanetworkopen.2024.3793

**Published:** 2024-03-26

**Authors:** Jordan Everson, Nathaniel Hendrix, Robert L. Phillips, Julia Adler-Milstein, Andrew Bazemore, Vaishali Patel

**Affiliations:** 1 Office of the National Coordinator for Health Information Technology, Washington, DC; 2 American Board of Family Medicine, Lexington, Kentucky; 3 Center for Professionalism and Value in Health Care, Washington, DC; 4 Division of Clinical Informatics and Digital Transformation, Department of Medicine, University of California, San Francisco

## Abstract

**Importance:**

Enabling widespread interoperability—the ability of health information technology systems to exchange information and to use that information without special effort—is a primary focus of public policy on health information technology. More information on clinicians’ experience using that technology can serve as one measure of the impact of that policy.

**Objective:**

To assess primary care physician perspectives on the state of interoperability.

**Design, Setting, and Participants:**

A cross-sectional survey of family medicine physicians in the US was conducted from December 12, 2021, to October 12, 2022. A sample of family medicine physicians who completed the Continuous Certification Questionnaire (CCQ), a required part of the American Board of Family Medicine certification process, which has a 100% response rate, were invited to participate.

**Main Outcomes and Measures:**

Eighteen items on the CCQ assessed experience accessing and using various information from outside organizations, including medications, immunizations, and allergies.

**Results:**

A total of 2088 physicians (1053 women [50%]; age reported categorically as either ≥50 years or <50 years) completed the CCQ interoperability questions in 2022. Of these respondents, 35% practiced in hospital or health system–owned practices, while 27% practiced in independently owned practices. Eleven percent were very satisfied with their ability to electronically access all 10 types of information from outside organizations included on the questionnaire, and a mean of 70% were at least somewhat satisfied. A total of 23% of family medicine physicians reported information from outside organizations was very easy to use, and an additional 65% reported that information was somewhat easy to use. Only 8% reported that information from different electronic health record (EHR) developers’ products was very easy to use compared with 38% who reported information from the same EHR developer’s product was very easy to use.

**Conclusions and Relevance:**

This survey study of family medicine physicians found modest and uneven improvement in physicians’ experience with interoperability. These findings suggest that substantial heterogeneity in satisfaction by information type, source of information, EHR, practice type, ownership, and patient population necessitates diverse policy and strategies to improve interoperability.

## Key Points

**Question:**

What were primary care physician perspectives on access to health information from outside organizations after years of policy support for interoperability (the ability of health information technology systems to exchange information and to use that information without special effort)?

**Findings:**

In this survey study of 2088 physicians, 70% indicated being at least somewhat satisfied with access to outside information. However, only 23% indicated that it was very easy to use outside information, and very few (8%) indicated that it was very easy to use information from different electronic health record systems.

**Meaning:**

The findings of this study suggest that 6 years after the 21st Century Cures Act, the policy goal of ubiquitous, high-value interoperable health information appears to remain a work in progress with heterogeneous barriers—including both too much information and missing information—limiting the value of interoperability.

## Teaser

This survey study examines the ease of use by US family medicine physicians in obtaining information on patients through electronic health records, both within and across varied developers.

## Introduction

Starting in 2016 with the 21st Century Cures Act and concurrent changes to the Merit-Based Incentive Payment System, public policy on health information technology focused more directly on interoperability, which is the ability of health information technology (IT) systems to exchange information between systems and use that information without special effort.^1^ The core regulations from the Cures Act intended to improve interoperability are now in place, with central applicability dates in 2021 and after. These include efforts to raise the bar for investment in interoperability (with some provisions applicable April 2021),^2^ create stronger financial disincentives for impeding interoperability (applicable as of April 2021),^2,3^ improve data standards to enhance the usability of exchanged information (with some provision applicable as of January 1, 2023),^2^ improve electronic health record (EHR) capabilities to allow outside applications to access EHR data (with some provisions applicable as of January 1, 2023),^2^ and create a universal floor for nationwide health information exchange (launched December 2023).^4^ These efforts, along with ongoing federal support for networks that help federally qualified health centers use health IT^5^ and efforts to enable bidirectional exchange of public health information^6^ present a broad attempt to increase investment in interoperability and support the effective use of health information to care for patients.

Available data indicate that these recent policy levers are needed. According to a nationally representative survey of outpatient physicians, even as recently as 2021, only 16% indicated that they engaged in 4 domains of interoperable exchange: electronically sending, receiving, searching for, and integrating information into their EHR, which is far below similar measures among hospitals.^7,8^ More granular information on physicians’ experience accessing and using information from outside organizations, which effective interoperability should improve, could provide insight into areas where interoperability currently supports the delivery of care and where challenges persist; identify prominent barriers to achieving value from interoperable information and the fitness of ongoing policymaking to address those barriers; and indicate targeted objectives for future policymaking.

Effective interoperability is particularly important for primary care physicians (PCPs) because they serve as first contact to the health care system, provide continuity of care over time, and have the responsibility for coordinating their patients’ care.^9,10,11^ More than one-third of all health care visits occur in primary care settings and more than 80% of people in the US have health care relationships in primary care.^12^ The ability to easily access and use information about patients from diverse sources is critical to PCPs, and PCPs have described interoperability as the most important improvement they wish to see in EHRs over the long term.^13^

We therefore sought to generate novel, robust measures of PCP’s experiences with interoperability that captured several dimensions and can serve as markers of progress on interoperability. We used an annual survey of family physicians to describe their (1) experience accessing and using information from outside organizations and variation by physician practice characteristics, (2) satisfaction with outside information by information type, and (3) variation in satisfaction by whether information came from the same or different EHR. These survey data are notable in that they accrue a 100% response rate from a nationally representative sample of family medicine physicians by being included as part of an annual certification requirement, avoiding response bias present in other surveys of physicians.^14,15^

## Methods

### Data Source

The American Board of Family Medicine (ABFM) administers a Continuous Certification Questionnaire (CCQ) among participants who provide direct patient care. The CCQ is a required part of the ABFM certification process for more than 100 000 family physicians and has a 100% response rate. Family physicians take this web-based survey on a rolling basis every year based on their initial year of certification, offering a nationally representative sample. The CCQ is designed to help the ABFM understand changes in family physician practice relevant to training, certification, and competency to fulfill its business requirements and capacity to inform policies that affect practice. These data are secondarily valuable for research and are often used to study and inform understanding about practice. Questions were developed in collaboration between the ABFM, the Office of the National Coordinator for Health Information Technology, and researchers at the University of California, San Francisco. We developed and revised the questionnaire items and response options based on cognitive interviews with family physicians and conducted quality checks when programming the questions on the CCQ following recommendations by the American Association for Public Opinion Research (AAPOR) reporting guideline. A core set of questions was answered by all ABFM diplomates in the 2022 certification cohort (from December 12, 2021, to October 12, 2022), but most questions relevant to this study were randomly given to rotating panels to reduce overall question burden. This study followed relevant portions of the Strengthening the Reporting of Observational Studies in Epidemiology (STROBE) reporting guideline and the AAPOR reporting guideline. This study was approved by the institutional review board of the American Academy of Family Physicians, with a waiver of informed consent for this research because of the use of secondary data.

### Measures

In total, 18 items on the 2022 CCQ assessed specific dimensions related to family physicians’ experience accessing and using information from outside organizations, a crucial marker for how interoperable technology is shaping the delivery of health care as experienced by clinicians. The questionnaire is included in the eAppendix in Supplement 1.

The CCQ included 10 items about respondents’ satisfaction with electronically accessing different types of clinical information from organizations outside their health system. For each item, respondents could indicate that they did not have or use it, or were not at all, somewhat, or very satisfied with accessing each type of information.

The CCQ included 4 items to capture respondents’ experience using information accessed from outside organizations. These items focused on the ease of using outside information to effectively care for patients, the ease of finding specific information, and the ease of using information from outside organizations that used EHR products from the same or different developer.

Four items on the CCQ assessed the prevalence of specific barriers related to using information from outside organizations accessed via EHRs. The barriers included not having a patient record available, missing key information within the record, difficulty finding important information due to a large amount of low-value information, and information was not integrated within the EHR.

Questions related to physicians’ experience and specific barriers to using information from outside organizations were not focused solely on information exchanged via electronic methods but rather were intended to capture a broader assessment of the ease of using information, which still often relies on telephone, fax, and other means.

### Statistical Analysis

We first assessed the distribution of responses by each information type individually and then derived a composite scale based on the 10 satisfaction-related items by assigning a value of 0 to each item that respondents did not have, did not use, or were not at all satisfied with because both responses represented no value to the clinician; a value of 1 to items respondents were somewhat satisfied with, and a value of 2 to items respondents were very satisfied with. The resulting scale ranged from 0 to 20. After calculating scale reliability (Cronbach α=0.96), we used this scale to calculate the empirical cumulative density function of respondents’ satisfaction accessing information from outside organizations overall. The resulting percentages can be interpreted as the percentage of respondents with some minimum level of satisfaction (for instance, at least somewhat satisfied with all items) or an equivalent score.

Based on examining the cumulative density function described, we used a multivariable logistic regression model with a binary outcome indicating whether respondents were very satisfied with at least 5 information types, representing approximately the top quarter of physicians. This model examined the association between the physician’s practice characteristics, EHR developer, and satisfaction with access to external information. Model coefficients are reported as odds ratios (ORs) with associated 95% CIs.

We next tabulated respondents’ experience using outside information, including the ease of using information from outside organizations to provide beneficial care for patients, the ease of finding specific information, and the ease of using information from outside organizations. We stratified respondents’ answers to questions about ease of using information from the same and different developers by the respondent’s developer. We then similarly tabulated their experience of the 4 barriers described earlier. We tested for differences in response patterns to ordinal survey items using χ^2^ tests; the data were not paired. Statistical significance was set at *P* < .01. Statistical analysis was conducted using Stata SE, version 15 (StataCorp LLC).

## Results

A total of 2088 family physicians (1053 women [50%]; 1035 men [50%]; age was reported categorically as either ≥50 years or <50 years) received and responded to the interoperability questions (100% response rate). Of these respondents, 27% practiced in independently owned medical practices, 35% practiced in hospital or health system–owned practices, and the remainder practiced in a variety of settings (eTable in Supplement 1). A total of 42% of respondents practiced in principal practice sites with between 1 and 5 clinicians. A total of 24% of respondents indicated that more than half of the patient population was part of a vulnerable group (ie, defined in survey question as uninsured, receiving Medicaid, homeless, low income, non-English speaking, racial and ethnic minority, or otherwise traditionally underserved group). Because respondents were required to complete the CCQ, there were no missing responses to individual items.

**Figure 1.**
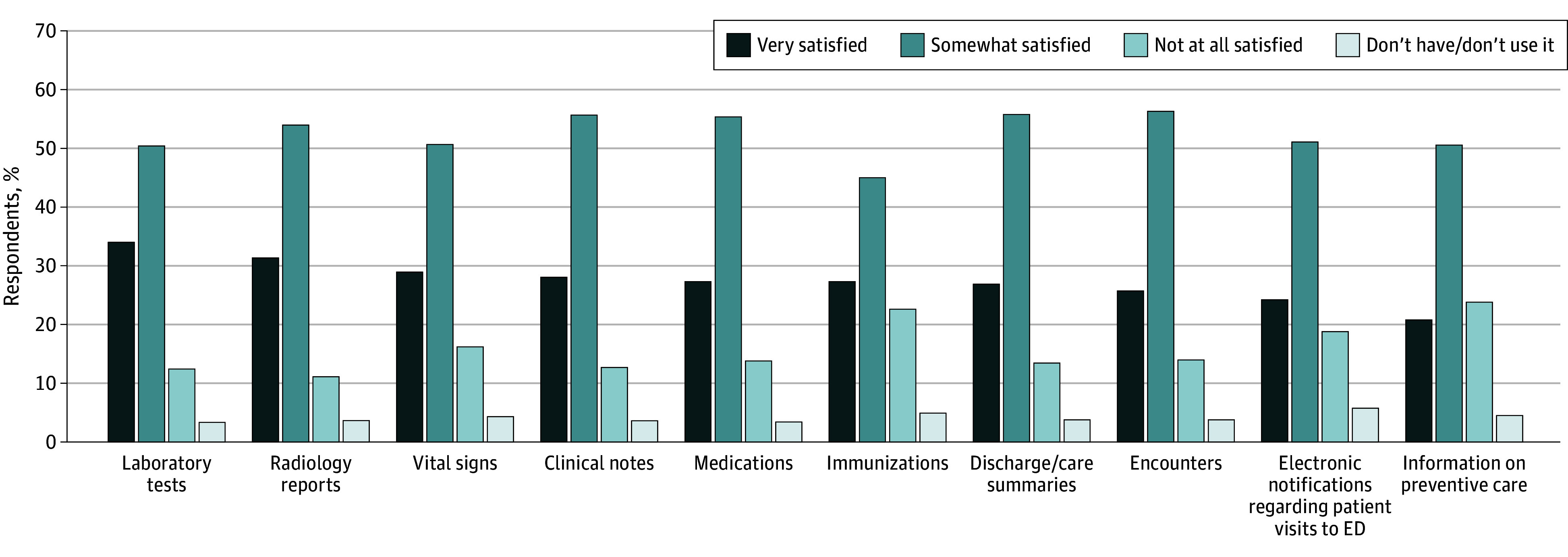
Family Physician Satisfaction With Access to External Patient Information, by Information Type Response to 2022 American Board of Family Medicine Continuous Certification Questionnaire by 2088 physicians. ED indicates emergency department.

Satisfaction with electronic access to information varied notably by data type (Figure 1). A total of 34% of respondents indicated that they were very satisfied with their ability to access laboratory information from external organizations—this was the highest reported rate. The lowest reported rate of satisfaction was for information on preventive care: 21% of respondents indicated they were very satisfied with their ability to access that information.

Five percent of respondents reported either not having or not using access to outside immunization information, and an additional 23% were not at all satisfied with their access to it. Similarly, 5% of respondents did not have access to preventive care information and 24% of respondents were not satisfied with their access to information on preventive care. The next highest rate of dissatisfaction was reported for access to electronic notifications regarding patient visits to emergency departments, with 6% of respondents indicating that they either did not have or did not use this information, and another 19% saying that they were not at all satisfied with their access to that information.

**Figure 2.**
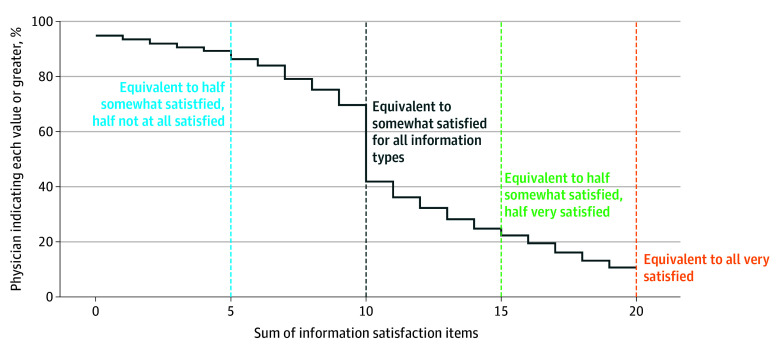
Cumulative Distribution of Family Physician Satisfaction With Access to External Information Response to 2022 American Board of Family Medicine Continuous Certification Questionnaire by 2088 physicians.

The composite scale of physician satisfaction with electronic access to outside information across information types indicated substantial variation in PCP satisfaction as evident by the slope of the empirical cumulative distribution function (Figure 2). A mean of 70% of respondents reported a composite score equivalent to at least somewhat satisfied with all information types or better (with 13% of respondents indicating exactly somewhat satisfied on all items). Twenty-five percent of respondents reported a composite score equivalent to half somewhat satisfied and half very satisfied or better, 11% of respondents indicated that they were very satisfied with access to outside information for all information types, and 11% of respondents indicated that they were not at all satisfied with their access to half or more of the information types.

Family physicians who treated a large proportion of vulnerable patients (OR, 0.75; 95% CI, 0.58-0.99) (Table) or did not have staff and linkages to community programs to address patients’ social needs (OR, 0.52; 95% CI, 0.40-0.66) were less likely to report being very satisfied with their access to outside information, as were family physicians working at practices with more than 20 clinicians (OR, 0.68; 95% CI, 0.53-0.87). Family physicians who used Epic or Allscripts EHR products were substantially more likely than those using eClinicalWorks, the reference category, to be satisfied with access to external information (Epic: OR, 1.77; 95% CI, 1.25-2.51 and Allscripts: OR, 1.73; 95% CI, 1.06-2.81).

**Table.** Association Between Physician Satisfaction With Access to Information From Outside Organizations and Physician, Practice, and Developer Characteristic^a^

Characteristic	Very satisfied with access to at least half of information types, OR (95% CI)
Gender	
Female	1 [Reference]
Male	0.85 (0.64-1.14)
Region	
Midwest	1 [Reference]
Northeast	0.87 (0.64-1.18)
South	0.91 (0.72-1.17)
West	0.78 (0.60-1.03)
Missing	1.41 (0.49-4.05)
Age, y	
≥50	1 [Reference]
<50	0.74 (0.62-0.90)
Location	
Urban	1 [Reference]
Rural	0.75 (0.56-1.01)
Not provided	0.78 (0.37-1.61)
Principal practice ownership	
Hospital or health system–owned medical practice	1 [Reference]
Independently owned medical practice	0.67 (0.46-0.99)
Academic health center or faculty practice	0.86 (0.62-1.19)
Governmental	1.04 (0.78-1.37)
Other	1.24 (0.91-1.68)
Principal practice size	
1-5	1 [Reference]
6-20	0.75 (0.60-0.94)
>20	0.68 (0.53-0.87)
Percentage of your patient population in your principal practice site is part of a vulnerable group	
<10	1 [Reference]
10-49	0.99 (0.80-1.22)
≥50	0.75 (0.58-0.99)
Dedicated staff and linkages to community programs to address patients’ social needs	
Agree	1 [Reference]
Disagree	0.52 (0.40-0.66)
Neutral	0.69 (0.54-0.87)
Does your organization participate in 1 or more value-based care initiative(s)?	
Yes	1.14 (0.80-1.62)
I don’t know	1.27 (0.93-1.73)
Electronic health record developer	
eClinicalWorks	1 [Reference]
Allscripts	1.73 (1.06-2.81)
Cerner	1.48 (0.93-2.37)
Epic	1.77 (1.25-2.51)
NextGen	0.93 (0.55-1.57)
athenahealth	1.24 (0.82-1.88)
Other	1.03 (0.73-1.44)

Abbreviation: OR, odds ratio.

^a^ Total of 2031 observations.

**Figure 3.**
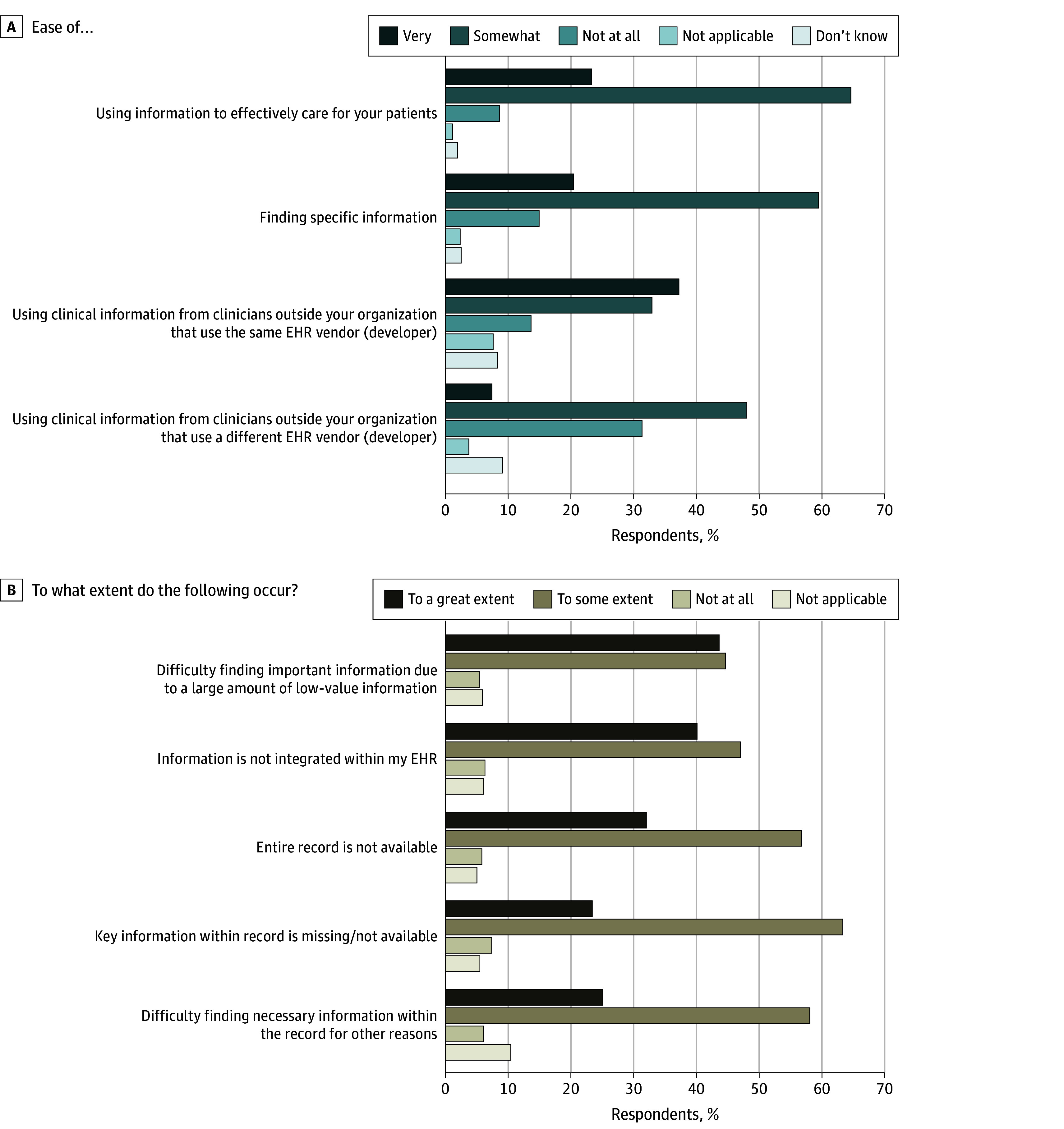
Family Physician Experience Using Outside Information Response to 2022 American Board of Family Medicine Continuous Certification Questionnaire by 2088 physicians. A, Ease of access to information in the electronic health record (EHR). B, Difficulties using the EHR.

When asked about the ease of using information when it was available, 23% of family physicians reported that it was very easy to use outside information to effectively care for their patients, 65% indicated it was somewhat easy to use, and 9% indicated it was not at all easy to use (Figure 3). Reponses were somewhat less positive when physicians were asked about the ease of finding specific information from external organizations: 20% of family physicians indicated it was very easy to find specific information, 59% indicated it was somewhat easy, and 15% indicated it was not at all easy to find specific information. Reported ease of using information overall was significantly different than ease of finding specific information (χ^2^ test *P* < .001). Thirty-eight percent of respondents indicated that information from outside organizations that used the same EHR was very easy to use. In contrast, 8% of respondents indicated that information from outside organizations that used a different EHR was very easy to use. Reported ease of using information from the same EHR was significantly different from ease of using information from a different EHR (χ^2^ test: *P* < .001).

When asked how often they encountered specific barriers, a substantial proportion of physicians indicated some level of missing information or difficulty finding information. Forty-four percent of physicians indicated a great extent of difficulty finding important information due to a large amount of low-value information (vs experiencing this to some extent or not at all), 40% indicated that information was not integrated into their EHR to a great extent, 32% indicated that the entire patient record was not available to a great extent, and 23% indicated that key information was missing within the patient's record to a great extent (Figure 3). In addition, 6% to 7% of respondents indicated not at all encountering these barriers.

**Figure 4.**
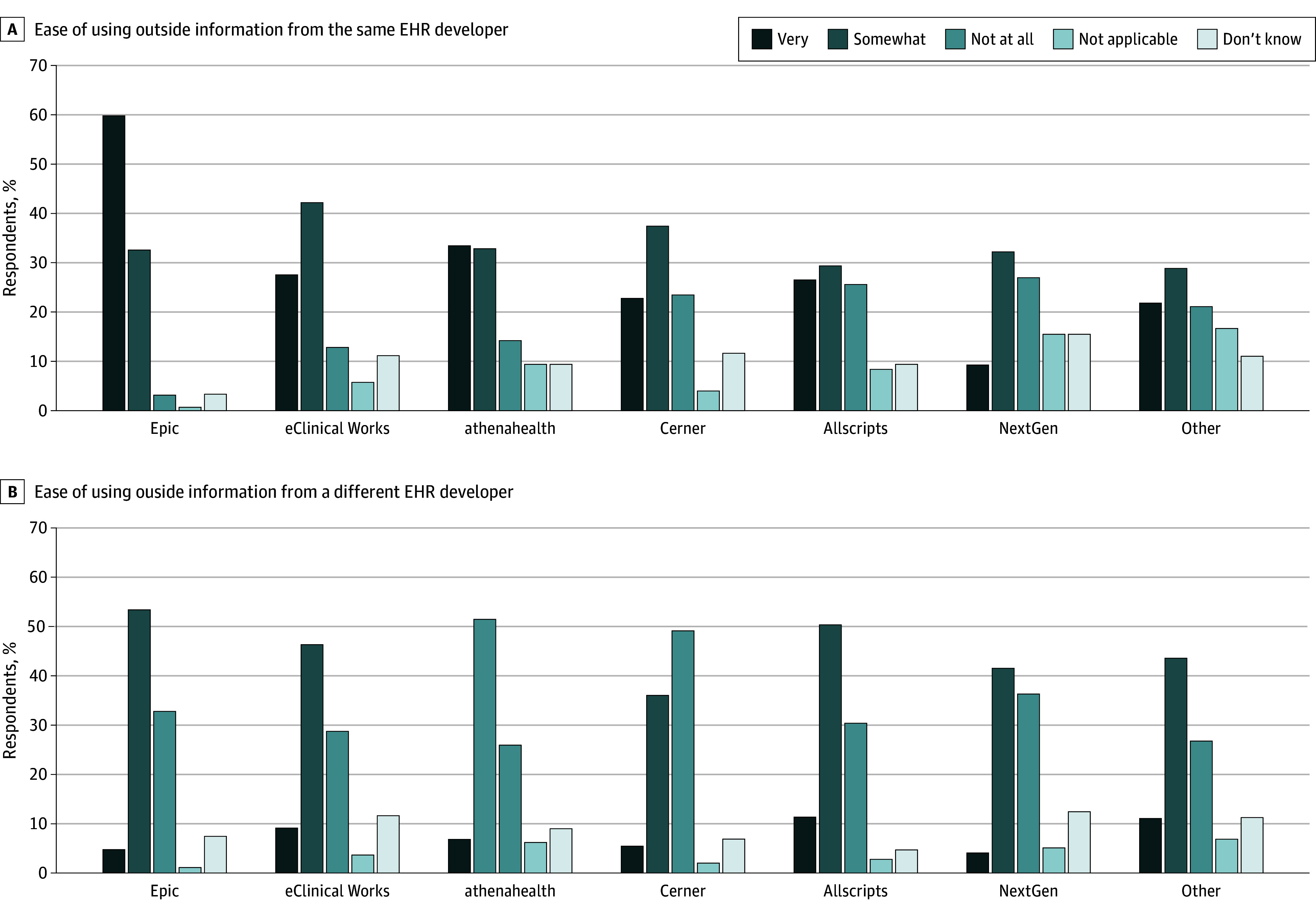
Ease of Using Outside Information From Electronic Health Record (EHR) Developers Response to 2022 American Board of Family Medicine Continuous Certification Questionnaire by 2088 physicians. Ease of using access to the same (A) or different (B) developers.

Rates of reported ease of using information from the same and different EHR developers varied substantially by EHR developer (Figure 4). Sixty percent of physicians using Epic indicated that information from outside organizations that used the same developer was very easy to use, as did 28% of respondents using eClinicalWorks and 34% of respondents using athenahealth. No more than 11% of respondents indicated that information from outside organizations using different EHR developers was very easy to use.

## Discussion

Primary care physicians have a critical need for information about their patients from all health care professionals, settings, and over time to fulfill their role in care integration, sense-making, and coordination. Nationally representative data on family physicians’ experience accessing and using information from outside organizations highlighted substantial but incomplete access to external information and moderate ease of using that information along with substantial variation in physicians’ experiences. Taken together, these data suggest that policy supporting interoperability has facilitated substantial but uneven progress in sharing information between organizations, and therefore numerous barriers must be addressed to ensure high-value access to interoperable information by clinicians working in a wide range of settings and with diverse technologies.

The composite findings related to satisfaction with access to information show substantial variation, with 11% of physicians reporting low satisfaction (ie, not at all satisfied with at least half of information types), just over one-quarter reporting high satisfaction (ie, very satisfied with at least half of information types), and 11% reporting that they were very satisfied with all information types. This variation highlights a challenge for initiatives aiming to improve information access for physicians because physicians at different points of satisfaction may have different interoperability needs or benefit from different initiatives to improve interoperability. For example, physicians who are highly dissatisfied—who we found to be more likely to serve vulnerable populations and to lack the resources necessary to address social needs (Table)—likely receive very limited or incomplete information from outside organizations and might benefit from focused efforts to make it easier to join an initial exchange network. In contrast, physicians who are somewhat to very satisfied overall may benefit from initiatives that increase the scale of exchange, such as work to link disparate networks to fill in remaining information gaps, such as the Trusted Exchange Framework and Common Agreement (TEFCA), and efforts to make directories of health care professionals’ electronic addresses widely available so that other professionals more often know where to direct information.^16^ More satisfied physicians may not need initiatives aimed at making it easier to participate in their first exchange network, since they are already participants.

Variation in satisfaction with access to different information types also suggests the need to address specific interoperability use cases that support the role of PCPs. As one example, the statistic that 28% of PCPs either do not have access to or are not at all satisfied with their ability to access immunization information suggests that part of the ongoing Centers for Disease Control and Prevention Data Modernization Initiative may need to focus on both PCPs’ ability to use immunization information and the public health system’s capacity to ensure that immunization information is available to PCPs.^6^ As a second salient example, one-quarter of PCPs indicated either not having or not being satisfied with notifications related to emergency department visits, which represents somewhat greater dissatisfaction than other information types.^17^ This is despite the fact that the Medicare Condition of Participation requires hospitals to provide PCPs with these event notifications. A model for evolving this requirement could be the approach to information reconciliation during care transitions within the Medicare Promoting Interoperability Program, in which clinician action to reconcile information into the EHR is incentivized, rather than only the sending and receiving of information.

One clear challenge is that patient information is often missing: 32% of respondents indicated frequently missing the patient’s entire record. While some amount of missing information may be due to care processes that do not support routine communication between health care professionals, improved health IT can play an important role in addressing missing information. Recent and ongoing policies, including full implementation of the 21st Century Cures Act’s information blocking provisions and launch of the TEFCA, which is meant to facilitate data sharing between disparate health information exchange networks, have the potential to increase access to information.^4,18^

When information was present, family physicians indicated that it was frequently not easy to use. Policies aimed at supporting or requiring the adoption and use of data standards are one approach to enable greater usability of exchanged data. Starting in 2023, health IT certified by the Office of the National Coordinator for Health Information Technology, which is used by the vast majority of physicians, must support the exchange of data classes as defined in the US Core Data for Interoperability.^19^ The use of standards aims to ensure that information is widely available in a structure that the receiving EHR understands so that useful information can be extracted and displayed for user review and follow-up. Increased adoption of standards may enable industry-led solutions aimed at increasing the usability of information, such as Google’s CareStudio and Epic’s HappyTogether, which both aim to make information easier to find for clinicians but are only available to a select set of physicians. Ideally, public and private sector initiatives—potentially including the use of large language models, such as GPT-4 (OpenAI), to summarize and simplify sometimes bloated clinical documents—will continue to grow and complement each other to make rapid progress in the presentation of information to a broad set of users.^20,21,22^

In addition, to some degree it should not be surprising that PCPs report far greater ease of use of information from outside organizations that use the same, rather than a different, EHR developer. A previous investigation found objectively greater interoperability between sites using the same EHR developer,^23^ and the Epic Care Everywhere product is well known to offer useful connectivity between its customers.^24,25^ Although PCPs using Epic were the most likely to report information from outside organizations that used the same developer was easy to use, only 5% of Epic users indicated outside information from a different EHR was very easy to use. An important metric of success for policy efforts to support interoperability is parity in the reported ease of use of information from the same and different EHR developer’s product, and substantial efforts remain to achieve that parity.

### Limitations

Our study is subject to some limitations. Our study is focused on a single primary care specialty. It is possible that other PCPs have different experiences. Our results are also likely not reflective of the views of medical specialists and surgeons. Nonetheless, family physicians represent nearly a quarter of all outpatient visits and predominantly use the same EHRs as those in other specialties, suggesting that these results are likely generalizable. Second, these data are self-reported, and physicians’ recollections may be biased. However, cognitive interviews indicated that physicians clearly understood the questions asked and were able to address them directly.

## Conclusions

In this survey study of family physicians, respondents described substantial but uneven satisfaction with their ability to access and use information from outside organizations to care for patients in their practices. Findings revealed that satisfaction varied across multiple dimensions—by physician, by the EHR from which the data originated, and by the type of information—and that physicians frequently experienced both missing information and too much information. Taken together, these data suggest a need for diverse and targeted approaches to complete progress toward universal, high-value interoperability.

## Notes

Supplement 1. **eTable.** Characteristics of the Sample **eAppendix.** 2022 ABFM CCQ Health IT Questions

Supplement 2.
Data Sharing Statement
