# Supplementary material for: Primary Care Physicians’ Satisfaction With Interoperable Health Information Technology
Source: JAMA Netw Open. 2024 Mar 26;7(3):e243793. doi: 10.1001/jamanetworkopen.2024.3793 (PMC10966410; doi:10.1001/jamanetworkopen.2024.3793)
Supplement: Supplement 1. — eTable. Characteristics of the Sample eAppendix. 2022 ABFM CCQ Health IT Questions [file jamanetwopen-e243793-s001.pdf]

Supplementary Online Content

Everson J, Hendrix N, Phillips RL, Adler-Milstein J, Bazemore A, Patel V. Primary care physicians' satisfaction with interoperable health information technology. *JAMA Netw Open*. 2024;7(3): e243793. doi:10.1001/jamanetworkopen.2024.3793

eTable. Characteristics of the Sample

eAppendix. 2022 ABFM CCQ Health IT Questions

This supplementary material has been provided by the authors to give readers additional information about their work.

© 2024 Everson J et al. *JAMA Network Open.*

eTable. Characteristics of the Sample

Characteristic	N	%	Characteristic	N	%
**Gender**			**Percent of your patient population in your principal practice site is part of a vulnerable group**		
Women	1,053	50			
Men	1,035	50	<10%	903	43
**Region**			10-49%	691	33
Midwest	507	24	>50%	494	24
Northeast	290	14	**Dedicated staff and linkages to community programs to address patients' social needs**		
South	733	35			
West	534	26	Disagree	419	20
Missing	24	1	Agree	1,229	59
**Age**			Neutral	440	21
50+	843	40	**Does your organization participate in one or more value-based care initiative(s)?**		
<50	1,245	60			
**Location**			No	257	12
Urban	1,765	85	Yes	1,409	67
Rural	273	13	I don't know	422	20
Missing	50	2			
**Principal Practice Ownership**			**Electronic Health Record Developer**		
Hospital / health system owned medical practice	735	35	eClinicalWorks	239	11
Independently owned medical practice	567	27	Allscripts	105	5
Academic health center / faculty practice	158	8	Cerner	144	7
Governmental	330	16	Epic	769	37
Other	298	14	NextGen	96	5
**Principal Practice Size**			athenaHealth	188	9
1-5 Providers	873	42	Other	547	26
6-20 Providers	658	32			
>20 Providers	557	27			

© 2024 Everson J et al. *JAMA Network Open.*

eAppendix. 2022 ABFM CCQ Health IT Questions

(1) Does your organization participate in one or more value-based care initiative(s), such as a patient centered medical home, accountable care organization or pay-for-performance arrangement?
□1 Yes
□3 No
□4 Don't Know

(2) What is the name of your current PRIMARY, outpatient EHR system? CHECK ONLY ONE BOX. IF OTHER IS CHECKED, PLEASE SPECIFY THE NAME.
□1 Allscripts
□2 athenahealth
□3 Cerner
□4 eClinical Works
□5 e-MDs
□6 Epic
□7 Modernizing Medicine
□8 NextGen
□9 Practice Fusion
□10 Greenway
□11 Other, specify: ____________________
□12 Unknown
□13 None

(3) How long have you used your current primary, outpatient EHR system?
____YEARS, ____ MONTHS

(4) Overall, how satisfied are you with your current primary, outpatient EHR system?
□1 Very dissatisfied
□2 Somewhat dissatisfied
□3 Neither satisfied nor dissatisfied
□4 Somewhat satisfied
□5 Very satisfied
□6 Not applicable

© 2024 Everson J et al. *JAMA Network Open.*

(5A) When you access clinical information from <u>outside</u> your organization (e.g. referrals, consult notes, discharge summaries, patient records) through any means (e.g. fax, phone, EHR, etc), how easy is it to use the information to effectively care for your patients?
□1 Not at all
□2 Somewhat
□3 Very
□4 Not Applicable
□5 Don't Know

(6A) When you access clinical information about your patients from <u>outside</u> your organization (e.g. referrals, consult notes, discharge summaries, patient records), how often is it…

	Never<br>1	Rarely<br>2	Sometimes<br>3	Often<br>4	Don't Know<br>5
available as a scanned document?					
in an electronic portal (e.g. to a health information exchange) separate from your EHR?					
from within your EHR in any integrated format (as opposed to a PDF)?					

(7A) When you access clinical information from <u>outside</u> your organization <u>in your EHR</u>, how easy is it to find specific information (e.g., medications, vitals, procedures, lab results)?
□1 Not at all
□2 Somewhat
□3 Very
□4 Not Applicable
□5 Don't Know

(8A) How easy is it to use clinical information from clinicians outside your organization that…

	Not at all<br>1	Somewhat<br>2	Very<br>3	Not Applicable<br>4	Don't know<br>5
Use the same EHR Vendor					
Use a different EHR Vendor					

(9A) When looking for or using clinical information from <u>outside</u> your organization, to what extent do the following occur:

	Not at All<br>1	To Some Extent<br>2	To a Great Extent<br>3	Not Applicable<br>4
Entire record is not available				
Key information within record is missing/not available				
Information is not integrated within my EHR				
Difficulty finding important information due to a large amount of low-value information				

© 2024 Everson J et al. *JAMA Network Open.*

Difficulty finding necessary information within the record for other reasons				

(10A) Rate your current satisfaction with accessing the following types of external patient information electronically (within your EHR and/or portal).

	Not at All Satisfied 1	Somewhat Satisfied 2	Very Satisfied 3	Don't Have/use it 4
Prior encounters (e.g., hospitalizations, ED visits)				
Clinical notes				
Labs				
Vitals				
Immunizations				
Radiology reports				
Information on preventative care (e.g., last mammography date)				
Discharge/care summaries				
Electronic notifications regarding patient visits to Emergency Department				
Medications				

(11A) Rate your sense of the general importance of accessing the following types of external patient information electronically (within your EHR and/or portal).

	Not at All Important 1	Somewhat Important 2	Very Important 3
Access to clinical notes from other health systems/organizations			
Access to medications from other health systems/organizations			
Receiving electronic notifications regarding patient visits to Emergency Department			
Access to social determinants of health information (e.g. housing stability, food insecurity) from other health systems/organizations			
Access to prior authorization determination from insurers			
Access to patient-specific medication prices and alternatives from insurers			

© 2024 Everson J et al. *JAMA Network Open.*

(5B) Over the past three months, what percent of your patient visits are delivered via telemedicine?
□1 None
□2 Less than 25%
□3 25% to 49%
□4 50% to 74%
□5 75% or more
□6 Don't know

(6B) On average, how many hours per day do you spend outside of normal office hours documenting clinical care in your current, primary outpatient EHR system?
□1 None
□2 Less than 1 hour
□3 1 to 2 hours
□4 3 hours to 4 hours
□5 More than 4 hours

(7B) To what extent does the need to go to multiple different sources (including providers, payers, and others) outside your primary EHR to access clinical information on your patients result in unnecessary burden?
□1 Not at All
□2 To some extent
□3 To a great extent
□4 To an excessive extent

(8B) Please indicate whether you use any of the following resources to help reduce time that you spend documenting in the EHR.

	Yes, and it leads to reduced time 1	Yes, but it doesn't reduce time 2	No 3
Scribe (any model)			
Support from other staff (e.g. medical assistants, nurses)			
EHR templates			
Voice recognition/transcription			

(9B) How would you assess the following usability dimensions of your current primary EHR system?

	Poor 1	Fair 2	Good 3	Excellen t 4	N/A 5
Ease of entering information					
Readability of information					
Amount of information presented on each screen					

© 2024 Everson J et al. *JAMA Network Open.*

Alignment with your workflow/cognitive process					
Relevant information is easy to find					
Usefulness of alerts					

(10B) Does your primary outpatient EHR electronically…

	Yes 1	No 2	Don't know 3	Not Available 4
report immunization data to your State's immunization information system (IIS)?				
access immunization data from your State's IIS?				

(11B) How are you able to view immunization data from outside of your organization? Check all that apply.

□1 In primary outpatient EHR
□2 In an electronic portal outside the EHR (e.g. to an IIS or Health Information Exchange)
□3 Other (e.g., paper or fax)
□4 I can't view these data
□5 Don't know

(12B) How often do you document screening for social needs (such as transportation, housing, food insecurity) in your primary outpatient EHR …

	Never 1	Rarely 2	Sometimes 3	Often 4	Don't know 5
by checking a box/button within the EHR?					
by writing it in a note?					
by entering it as a diagnosis (i.e. ICD-10-CM Z codes)?					

(1MU) How often do you electronically <u>send</u> patient health information to others outside your organization through your primary outpatient EHR system (not eFax) or via a Web Portal (separate from your EHR)?

☐1 Never
☐2 Rarely
☐3 Sometimes
☐4 Often
☐5 Don't know

(2MU) How often do you electronically <u>receive</u> patient health information from outside your organization using your primary outpatient EHR system (not eFax) or a Web Portal (separate from EHR)?

☐1 Never
☐2 Rarely
☐3 Sometimes
☐4 Often
☐5 Don't know

(3MU) Does your primary outpatient EHR system integrate any type of patient health information received electronically (not eFax) without special effort like manual entry or scanning? *Note: This refers to situations when you electronically receive patient health information from external organizations.*

□1 Yes
□2 No
□3 Don't know
□4 Not applicable

(4MU) When seeing a new patient or a patient who has previously seen another clinician, how often do you <u>electronically</u> search or query for your patient's health information from sources outside of your organization through your primary outpatient EHR system or via a Web Portal? *Note: This could include via remote or view only access to other facilities' EHR or health information exchange organization.*

☐1 Never
☐2 Rarely
☐3 Sometimes
☐4 Often
☐5 Don't know

(5MU) When treating patients seen by clinicians outside your organization, how often do you or your staff have clinical information from those outside encounters electronically available in your primary outpatient EHR? *Note: Electronically available does not include scanned or PDF documents.*

☐1 Never
☐2 Rarely
☐3 Sometimes
☐4 Often
☐5 Don't know
☐6 I do not see patients outside my medical organization.

© 2024 Everson J et al. *JAMA Network Open.*
