# Supplementary material for: Primary Care Physicians’ Satisfaction With Interoperable Health Information Technology
Source: JAMA Netw Open. 2024 Mar 26;7(3):e243793. doi: 10.1001/jamanetworkopen.2024.3793 (PMC10966410; doi:10.1001/jamanetworkopen.2024.3793)
Supplement: Supplement 2. — Data Sharing Statement [file jamanetwopen-e243793-s002.pdf]

Data Sharing Statement

Everson. Primary Care Physicians' Satisfaction With Interoperable Health Information Technology. *JAMA Netw Open*. Published March 26, 2024.
doi:10.1001/jamanetworkopen.2024.3793

Data
Data available: No
